# Infrared Thermography of Cattle Hooves: Temperature Differences According to Lameness Score

**DOI:** 10.3390/ani16071058

**Published:** 2026-03-31

**Authors:** Tina Bobić, Mihaela Oroz, Marko Oroz, Nikola Raguž, Pero Mijić, David Kranjac, Mario Shihabi, Emmanuel Karlo Nyarko, Stanko Horvat, Boris Lukić

**Affiliations:** 1 Faculty of Agrobiotechnical Sciences Osijek, University of Josip Juraj Strossmayer of Osijek, 31000 Osijek, Croatia; mioroz@fazos.hr (M.O.); moroz@fazos.hr (M.O.); nraguz@fazos.hr (N.R.); pmijic@fazos.hr (P.M.); dkranjac@fazos.hr (D.K.); blukic@fazos.hr (B.L.); 2 Faculty of Agriculture, University of Zagreb, 10000 Zagreb, Croatia; mshihabi@agr.hr; 3 Faculty of Electrical Engineering, Computer Science and Information Technology, University of Josip Juraj Strossmayer of Osijek, 31000 Osijek, Croatia; karlo.nyarko@ferit.hr; 4 GAUSS Ltd., 31000 Osijek, Croatia; stanko.horvat@gauss.hr

**Keywords:** infrared thermography, lameness score, cattle hooves, temperature, coronary band

## Abstract

The aim of this study was to determine the distribution of and differences in the temperature values for the cows’ hooves depending on the lameness score and health status. The research was conducted on a free-stall housing dairy farm with *Holstein* cows (n = 180). The maximum (IR MAX), average (IR MEAN) and minimum (IR MIN) infrared temperature values were recorded at the coronary band (CB). The four-point manual locomotion scoring system was used to assess four levels of lameness score (LS0–LS3). The average values of IR MEAN, IR MIN and IR MIN of unhealthy hooves were significantly higher than the mean values of healthy hooves. Under lower ambient recording conditions, significant differences in CB temperature were observed between cows with different lameness scores, while under higher ambient conditions these differences were not statistically significant. The IR MEAN values for cows with LS0 (16.28 °C) were significantly (*p* < 0.05) lower than those for cows with LS1 (17.59 °C), LS2 (17.71 °C) and high significantly (*p* < 0.01) lower compared to cows with LS3 (18.04 °C). The distribution of the coronary band temperatures indicates that hoof temperature is associated with the health status of the cow, ambient recording conditions, and the level of the lameness score.

## Simple Summary

Lameness represents a major problem on dairy farms, with significant implications for animal welfare and farm profitability. Timely detection of hoof diseases and the consequent prevention of lameness is, therefore, crucial. There are several methods available for detecting lameness in cows, and these can be broadly classified as direct (cow gait analysis, image-processing techniques, weight distribution platforms, etc.) and indirect (sensors for movements, eating habits, lying times, etc.) approaches. The results of this research integrate the direct (lameness score) and indirect (infrared thermography) methods that will be the basis for further research aimed at connecting different diagnostic techniques to improve the reliability and safety of hoof disease detection and, thus, prevent lameness in cattle. The main findings of this research indicate that hoof temperature is associated with the health status of the cow, the ambient recording conditions, and the level of the lameness score. The main conclusion is that a combination of different methods increases the reliability and safety of hoof disease detection.

## 1. Introduction

Intensive dairy production faces numerous challenges, one of the most important being the high rate of lameness in dairy cows. Different studies have reported lameness rates ranging from 13% to 55% [1,2,3,4,5,6]. In addition to mastitis and reproductive problems, lameness is one of the most common issues on dairy farms [2,7,8,9]. According to Liang et al. (2017) [10], the cost of lameness in dairy herds varies considerably depending on the lesion type and severity, with estimated average losses ranging from USD 185 to USD 333 per case. Robcis et al. (2023) [11] estimated that the average annual cost per lameness case and per active case of digital dermatitis is approximately €308 and €392, respectively.

Depending on the authors, lameness has been defined as either an inability to use the limbs in an effective way or as dysfunctional use of the extremities [12,13,14,15]. Lameness has a negative effect on dairy herd performance and farm profitability due to its association with reduced milk yield [13,16], impaired reproductive performance [17] and an increased risk of culling [18]. In addition, lameness has a negative impact on the cows’ welfare, which is undesirable in modern production. Early detection of cows with a lower degree of lameness is, therefore, essential to prevent progression to more severe stages and to improve overall herd welfare.

There are several ways of detecting lameness, broadly classified as direct and indirect methods. According to ICAR [19], lameness scoring, when applied on a regular basis, allows the detection and treatment of lame cows at an early stage of the disease. One of the most widely used direct approaches involves monitoring and assessing a cow’s movement using a locomotion scoring system (mostly based on identifying asymmetric gait, weight distribution and an arched back posture) [14,20,21]. Among the indirect methods, infrared thermography (IRT) is commonly applied to monitor the temperature of specific body regions. The most common application of infrared thermography is the measurement of the hoof’s coronal temperature and that of the region between heel bulbs and the accessory digits [22]. Previous research has shown the effectiveness of IRT technology in detecting hoof diseases; for example, Álvarez et al. (2025) [23] found significantly higher temperatures in diseased compared to healthy cow hooves, and significantly different temperatures depending on the type of disease. Earlier studies by Alsaaod et al. (2015, 2019) [24,25] concluded that infrared technology as a non-invasive tool helps to detect the localization of areas of increased temperature, which may indicate inflammation, and can detect lameness and hoof diseases in dairy cows. The latest findings provided by Bumbálek et al. (2026) [26] indicate the possibility of automating IRT and deep learning systems on farms in the detection of diseases of the hooves and the prevention of lameness. Although both approaches have demonstrated good performance individually, their integration may enhance the reliability and accuracy of early lameness detection. Integrating different methods, particularly with the potential for automation, could significantly improve herd health monitoring and farm management.

The aim of this study was to determine the distribution and differences in hoof temperature values in dairy cows according to the lameness scores and hoof health status. The results provide the basis for further research focused on integrating different methods to improve the reliability of hoof disease detection and, thus, enhance the prevention of lameness under practical farm conditions.

## 2. Materials and Methods

The study was conducted on a free-stall housing dairy farm with straw bedding (45°40′33.6″ N, 18°38′27.6″ E, 83 to 90 m above sea level, with an average rainfall of 64 mm). For the purpose of this work, a period of 14 consecutive days (during October 2025) was selected. The study included one production group of *Holstein* cows (n = 180) that ranged from the 1st to 8th (mean = 2.98) lactation, and from 5 to 314 days in milk (mean = 81.35). The average daily milk yield of the group was 40.49 kg/day. The air humidity ranged from 73.5 to 86.60%, with an average value of 80.14% (Table 1).

### 2.1. Measurements

#### 2.1.1. Infrared Thermography

Hoof temperature measurements were performed using a thermal imaging camera (Thermal Advance Smart Sensor Camera, FLIR A700, FLIR, Wilsonville, OR, USA; IR lens F = 10 mm; 42° field of view; resolution 640 × 480; 30 Hz; temperature range −20 °C to 2000 °C). The camera was fixed at a distance of 0.50 m (emissivity = 0.95) from the concrete passage corridor used by cows after milking. The camera was placed under the roof and in the corner between two buildings, i.e., in the lee of the building, to avoid the direct influence of solar radiation and wind currents. The camera recorded all cows passing by after morning milking in real time and under farm conditions (cows and hooves were recorded without prior cleaning to remove dung, urine and other impurities). Animal identification was done manually by two trained operators, who recorded cow ID, the order in which each cow passed in front of the camera, and time of passing in front of the camera. The cow ID that was manually recorded by the observers was later merged with the IR data of the same cow during the analysis of the IR record and during the input of the results into the Excel database. The cow was identified based on the observer’s manual recording of the cow ID and the sequence and time record of the cow’s passage in front of the IR camera. Manual identification of cows was done because after milking, when leaving the milking parlor, the cows passed through the extended exit part of the milking parlor, where they were mixed together and did not exit in the exact order in which they entered for milking. Therefore, the data on cow ID and their milking order taken from the milking parlor were unusable. This disrupted their accurate identification when passing in front of the IR camera.

**Scheme 1:**
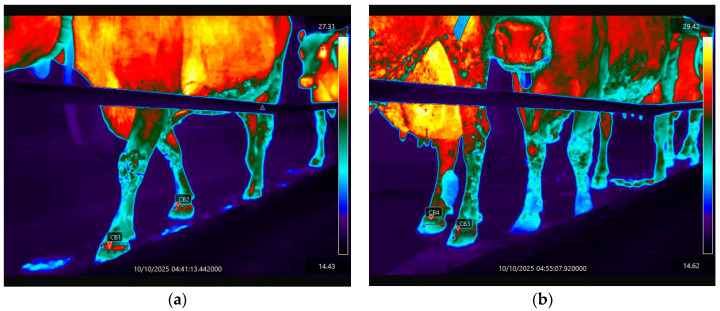
Thermovision analyses of video recordings. (**a**) Measuring the area of the coronary band (CB) of the front legs and (**b**) measuring the area of the coronary band (CB) of the hind legs.

The video recordings were processed using FLIR Research studio (Professional Edition) program. The maximum (IR MAX), average (IR MEAN) and minimum (IR MIN) infrared temperature values of the hooves were measured from the dorso-lateral aspect of the hoof at the level of the coronary band (CB), as shown in Scheme 1. Hooves that were freshly contaminated with cow feces or where the cow had stepped in fresh feces (which was easily visible on the recordings) were excluded from further analysis.

#### 2.1.2. Ambient Condition

Air temperature and relative humidity were measured using Extech RHT 20 data loggers. The devices were positioned in close proximity to the IR cameras. Measurements were recorded over the entire 14-day study period at one-hour intervals.

#### 2.1.3. Locomotion Score

The 4-point manual locomotion scoring system was used to assess the level of lameness in cows, according to the DairyNZ (2023) [27] guidelines, as presented in Table 2.

**Scheme 2:**
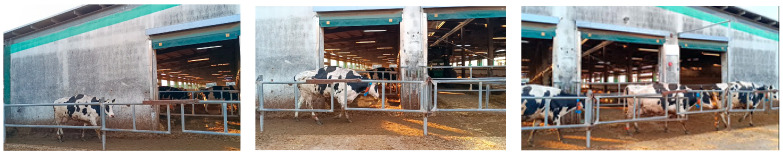
Manual locomotion scoring of the experimental cows.

Locomotion scoring was performed once during 14-day study period, immediately after thermovision recordings, in walking corridors as cows returned from milking to the barns (Scheme 2). Only one experienced observer assessed lameness.

#### 2.1.4. Examinations of the Hooves for Health Status

During the 14-day recording period, after locomotion scoring, all cows were isolated for hoof examination and health status determination. The examination was performed by an official and experienced hoof-trimming person in collaboration with a doctor of veterinary medicine. All four hooves per cow were examined. A hoof on which a disease was detected (ulcer, dermatitis, etc.) was classified as an “unhealthy” hoof, while a hoof on which no disease was detected was classified as a “healthy” hoof.

#### 2.1.5. Statistical Analyses

Statistical analysis of the data was performed using the STATISTICA software, (TIBCO Statistica, version 14.1.0.8. 2023) [28]. Prior to the application of parametric statistical tests, the normality of the data distribution was evaluated using the Shapiro–Wilk test. Homogeneity of variances was assessed using Levene’s test. Since the assumptions of normality and homogeneity of variances were satisfied, the analysis of variance (ANOVA) was applied to assess:

- the effect of hoof health status (healthy vs. unhealthy) on coronary band temperature values (IR MEAN, IR MAX, and IR MIN),
- the effect of lameness score levels on the temperature values (IR MEAN, IR MAX, and IR MIN) of the coronary band (CB) under different ambient conditions (lower ambient conditions: air temperature 7.40 °C and relative humidity 75.46% and higher ambient conditions: air temperature 14.27 °C and relative humidity 85.57%).

Post hoc comparisons were performed using Tukey’s HSD test, with the level of statistical significance set at *p* < 0.05.

## 3. Results

### 3.1. Thermovision

#### 3.1.1. Health Status of Cows’ Hooves

**Figure 1:**
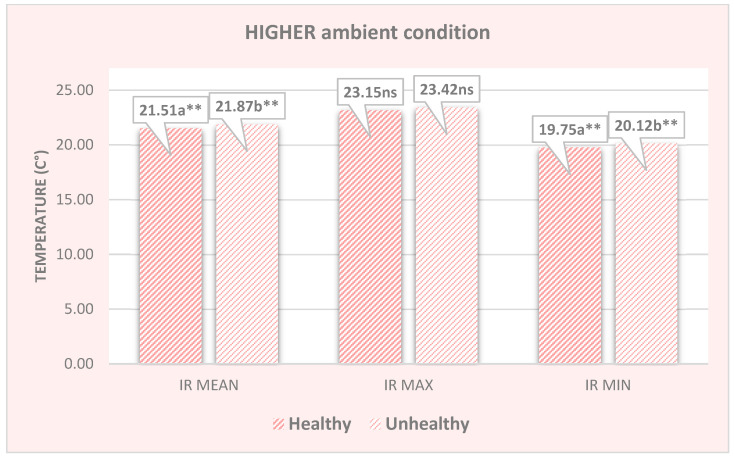
Temperatures of the coronary band for healthy and unhealthy hooves from infrared recordings in higher ambient conditions (ns = not significant; ** *p* < 0.01; ab = different letters between the columns of the chart indicate a significant difference).

Under higher environmental conditions (mean air temperature 14.27 °C and relative humidity 85.57%) significant differences in coronary band temperatures were found between healthy and unhealthy hooves. The mean IR MEAN and IR MIN values were significantly higher in unhealthy hooves compared to healthy hooves (IR MEAN: 21.87 vs. 21.51 °C, *p* < 0.01; IR MIN: 20.12 vs. 19.75 °C, *p* < 0.01; Figure 1). In contrast, IR MAX values did not differ significantly between healthy and unhealthy hooves (23.42 °C vs. 23.15 °C, *p* > 0.05; Figure 1).

**Figure 2:**
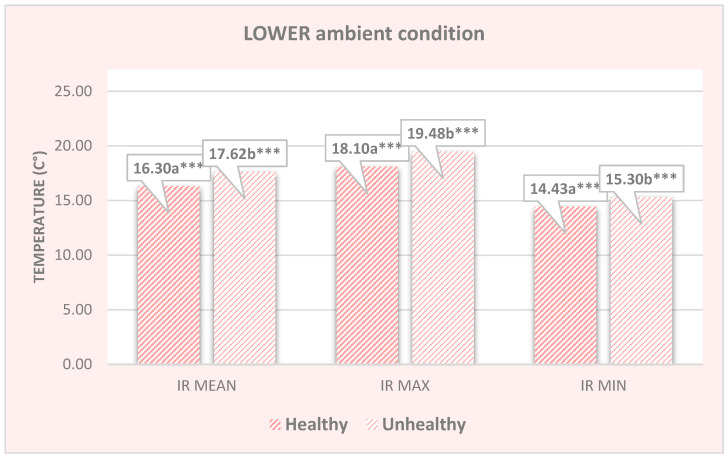
Temperatures of the coronary band for healthy and unhealthy hooves from infrared recordings in lower ambient conditions (*** *p* < 0.001; ab = different letters between the columns of the chart indicate a significant difference).

Under lower ambient recording conditions (mean air temperature 7.40 °C and relative humidity 75.46%), highly significant differences were found for all observed properties. In the coronary band region, the IR MEAN, IR MAX and IR MIN of unhealthy hooves (17.62, 19.48, 15.30, *p* < 0.001, Figure 2) were significantly higher compared with the same values for the coronary bands of healthy hooves (16.30, 18.10, 14.43, and *p* < 0.001, Figure 2).

#### 3.1.2. Locomotion Score

Under lower ambient recording conditions, significant differences in coronary band temperature were found between cows with different lameness scores, while under higher ambient conditions these differences were not statistically significant. The IR MEAN values for cows with LS0 (16.28 °C) were significantly lower (*p* < 0.05) than those for cows with LS1 (17.59 °C) and LS2 (17.71 °C), and significantly lower (*p* < 0.01) for cows with LS3 (18.04 °C), Table 3. A significant difference in IR MAX temperatures was found only between cows with LS0 and LS3 (17.98 vs. 20.02 °C, Table 3). IR MIN values of LS0 (13.87 °C) were significantly (*p* < 0.05) lower than for cows with LS3 (15.31 °C) and significantly lower (*p* < 0.01) than for cows with LS1 and LS2 (15.36 °C and 15.58 °C, respectively; *p* < 0.01), Table 3.

**Figure 3:**
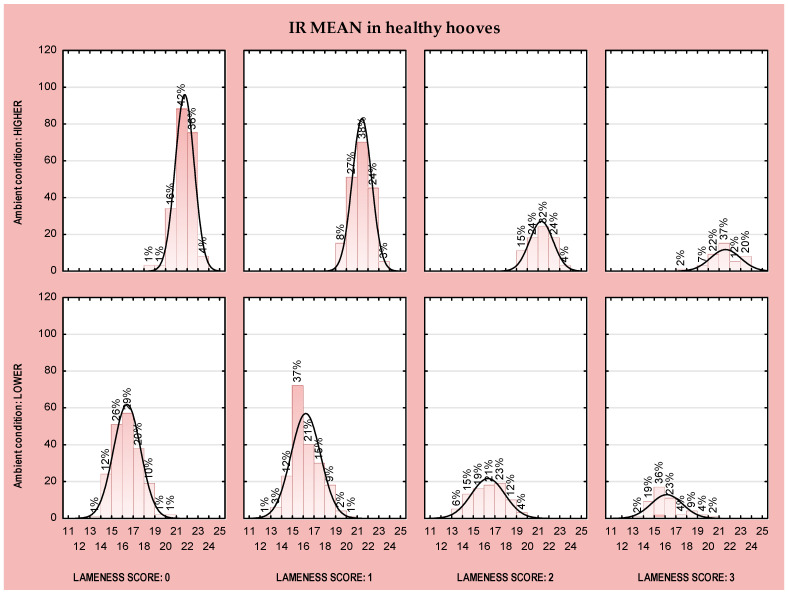
Distribution of temperature values of the IR MEAN in healthy hooves relating to lameness score and ambient conditions.

The distribution of coronary band (CB) temperatures indicated that hoof temperature was influenced by hoof health status, ambient recording conditions and lameness score. For example, under higher ambient recording conditions and considering only healthy hooves, the highest number of observed hooves (n) were from cows with a lameness score of (LS) 0 (n = 311) and 1 (n = 186). The majority of hooves had an IR MEAN temperature range of 20–21 °C (42%, LS0; 38%, LS1; 32%, LS2; and 37%, LS3, Figure 3), while the fewest hooves had temperatures lower than 20 °C (1%, LS0 and 2%, LS3, Figure 3).

Under lower ambient recording conditions, the distribution of CB temperatures in healthy hooves showed that the most frequently observed IR MEAN temperature range was 16–17 °C in cows with LS0 (29%, Figure 3), and 15–16 °C in cows with LS1 (37%, Figure 3). Only a small proportion of hooves exhibited IR MEAN temperatures below 14 °C (1%, LS0; 1%, LS1; and 2%, LS3, Figure 3) or above 19 °C (1%, LS0; 2% LS1; 4% LS2; and 4%, LS3, Figure 3). Regarding the total number of observed hooves, the highest number of hooves originated from cows with LS0 (n = 193) and LS1 (n = 186), which is similar to the results under higher ambient conditions (Figure 3).

**Figure 4:**
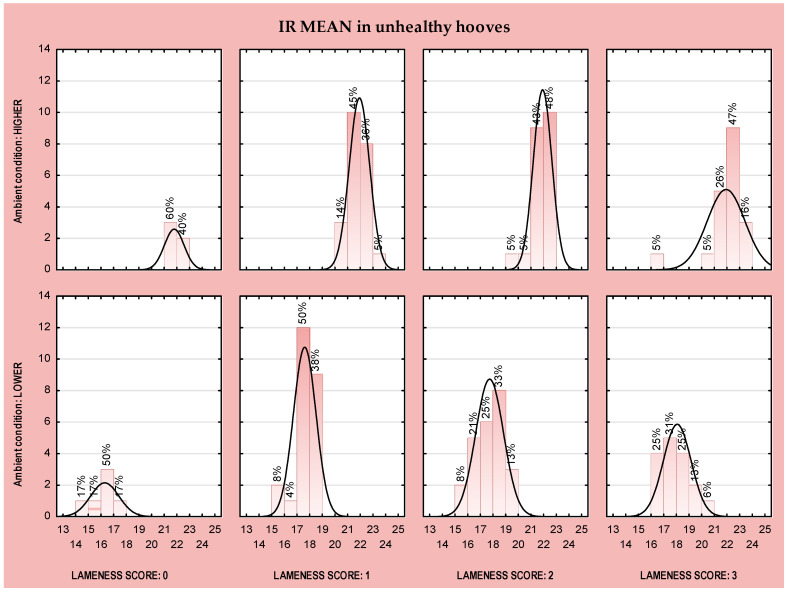
Distribution of temperature values of the IR MEAN in unhealthy hooves relating to lameness score and ambient conditions.

When analyzing IR MEAN temperature values in unhealthy hooves, the distribution pattern is completely different (Figure 4). Under higher ambient conditions, or when recording a very small number of observations, with fewer than four observed hooves, we recorded LS 0 with the temperature of their CB ranging from 20 to 21 °C and from 22 to 23 °C (n = 3, 60%; n = 2, 40%, Figure 4) and from 16 to 17 °C (n = 3, 50%, Figure 4), and when less than 16 °C (n= 2, 17%; Figure 4) or more than 17 °C (n = 1, 17%, Figure 4) in lower ambient conditions. The largest number of observed hooves had a temperature range of 21 to 22 °C (45%, LS1; 43% LS2; and 26%, LS3) and range from 22 to 23 °C (36%, LS1; 48% LS2; and 47%, LS3) (higher ambient conditions), and a temperature range of 17 to 18 °C (50%, LS1 and 31%, LS3) and range from 18 to 19 °C (38%, LS1 and 33% LS2) (lower ambient conditions).

## 4. Discussion

Lameness prevention is extremely important in intensive milk production, not only due to the economic losses [29] but also because of its impact on an animal’s welfare and their productive life [18]. Therefore, the use of various tools for disease detection is crucial and inevitable nowadays. The market offers a range of methods and tools that can improve the detection of hoof problems and support lameness prevention strategies [30,31]. Innovations such as infrared thermography are gaining an increasing volume of research in cattle production; thermal thresholds and temperature patterns that could facilitate the early detection of hoof lesions and inflammatory processes are being sought. The results obtained in this study provide another upgrade in the thermographic research field. A significantly higher temperature (up to 1 °C) in the coronal area of the diseased hooves compared to healthy hooves was recorded, confirming observations from earlier studies [24,32,33,34,35]. For example, Alsaaod and Büscher (2012) and Bobić et al. (2017) [32,33] confirmed that the surface temperature of the lame limb increases in the presence of hoof lesions. Wood et al. (2015) [36] concluded that feet with claw horn lesions generally exhibited higher temperatures than unaffected feet, with an average difference of approximately 0.393 °C. Moreover, feet with multiple lesions underwent a significantly higher temperature increase (approximately 2.005 °C), compared with lesion-free feet. These findings provide further evidence that localized temperature increases occur in the presence of inflammatory processes, and can be detected using infrared thermography. This observation is also supported by Cockcroft et al. (2000) [37], who concluded that thermography could identify the area of inflammation and can be used to identify septic arthritis in heifers. The results of this study also confirmed the strong correlation and influence of environmental temperature on infrared readings of hoof temperature. Coronary band temperatures differed substantially between lower and higher ambient conditions, irrespective of hoof health status. Under lower ambient temperatures, all hooves exhibited much lower IR values (by more than 5 °C) compared with measurements recorded with an IR camera under higher ambient conditions. Importantly, temperature differences between cows with different lameness scores were more pronounced under lower ambient conditions. This can also be referenced in the results of Church et al. (2014) [38], who found that airflow intensity lowered IR readings by 0.43 ± 0.13 °C at 7 km/h wind speed and by 0.78 ± 0.33 °C at 12 km/h wind speed. Likewise, reductions in air temperature of more than 6.5 °C, combined with changes in air velocity within the barn, have been shown to significantly decrease surface temperature readings in cattle [32]. According to Wood et al. (2015) [36], for every ±1 °C change from the average ambient temperature (13.6 °C), the foot temperature will change ±0.28 °C. Taken together, these findings and the results from this study confirm the high sensitivity of IRT measurements to ambient conditions (primarily air temperature and humidity, along with other things such as wind, direct sunlight, contamination from urine and feces, etc.). Therefore, environmental factors must be carefully considered when establishing temperature thresholds and when implementing IRT in automated systems for early detection of hoof disorders in dairy cows.

An important advancement highlighted in the present study is the connection between lameness score and hoof temperature values. Significant differences were observed in coronary band temperatures between cows with lower LS scores and those with higher LS values. This can be explained by the fact that during the onset of inflammation and changes in circulation in the inflamed part of the body, the temperature rises. In this context, the lameness score represents a useful indicator for the detection of hoof diseases. In combination with thermography, it is easier to localize inflammation and increase the certainty of detecting hoof disease, thus enabling the prevention of more severe stages of lameness. These results are consistent with Werema et al. (2023) [39], who reported that increases in mean foot skin temperature were associated with higher locomotion scores. However, not all studies have confirmed this relationship; for example, Renn et al. (2014) [40] did not detect significant differences between mean temperature values across all lameness categories, but they observed an association between individual foot temperatures over consecutive weeks and identified a high level of hind limb lameness when using a disease threshold of 27 °C. The same authors concluded that thermal imaging techniques provide a more objective and more reliable assessment compared with subjective locomotion scoring by human observers. Furthermore, Doğan et al. (2024) [41] did not determine any significant correlation between the lameness score and any specific region on the hoof or limb. However, they reported significant correlation between different anatomical regions of the hooves in relation to lameness severity. The same authors also concluded that thermography could serve as a guide for techniques like lameness scoring and could support preventive health management strategies.

The health status of the hooves in combination with the lameness score revealed a certain temperature distribution pattern (dispersion). The number of observed hooves and their corresponding temperature values reflected the lameness score category and the underlying health status. In healthy hooves, their number and temperature values were skewed to the left, i.e., towards lower lameness score values (LS0 and LS1 scores dominate compared to LS2 and LS3 scores), while in unhealthy hooves, the opposite results were obtained, which gravitated towards the highest lameness score values (LS1, LS2 and LS3 scores being more prevalent than LS0). The explanation of this pattern lies in the fact that each cow has four hooves, and in many cases only one or two may be affected by pathology. The presence of one or more diseased hooves can result in impaired locomotion and, consequently, a higher lameness score, even though some hooves of the same animal remain clinically healthy. Conversely, several cases were observed in which cows classified as LS0 exhibited localized inflammatory changes detected by thermography, suggesting that subclinical alterations may precede visible locomotor impairment. The majority of observations were concentrated within IR MEAN temperature range of 20 to 21 °C. However, cows with LS0 and LS1 showed greater dispersion of temperature values, whereas LS2 and LS3 categories displayed a more compact distribution, with a shift toward higher temperature ranges. As expected, the IR MEAN values of diseased hooves were consistently shifted toward higher temperatures compared with healthy hooves, which is in agreement with the earlier findings of Werema et al. (2021) [42], who said that with an increasing locomotion score the mean foot skin temperature also increased, which shows that infrared measurement of temperature could be a useful alternative to locomotion scoring. This is contrary to the findings of Rodríguez et al. (2016) [43], who concluded that IRT is not a practical method to be used on farms and does not contribute to early lameness detection, and who did not find significant temperature changes for cows from different breeds (*Friesian*, *Kiwi cross*, or *Jersey*) with lower lameness score (LS1 and LS2).

A previous study by Sousa et al. (2020) [44] confirmed the high efficiency of locomotion score (100% sensitivity, 97% specificity and 98% accuracy) and infrared thermography (96% sensitivity, 63% specificity, and 75% accuracy) in detecting laminitis in zebu cattle. They concluded that those two methods are good and non-invasive methods for detecting acute hooves inflammation in cattle.

Despite the challenges associated with the application of thermography, such as variability in temperature distribution depending on leg position, animal age, stage of production, and the strong influence of environmental conditions, the present study confirms the practical value and applicability of IRT. Clear differences were observed between healthy and diseased hooves, as well as between cows with different lameness scores. These findings are consistent with recent research by Bumbálek et al. (2026) [26], who integrated IRT with a deep learning-based system for automated detection and localization of hoof diseases under real farm conditions. Their results demonstrated a high hoof detection rate and reliable health classification performance. The authors emphasized that such systems enable early, non-invasive monitoring of hoof health and support practical implementation within precision livestock farming frameworks.

### Considerations and Limitations When Using Thermography in Lameness Detection

In this study, an elevated temperature in unhealthy hooves was determined and the association between elevated hoof temperature and an increase in the lameness score was also determined. The distribution of lameness scores showed that it is possible to find diseased hooves even in those cows that have a perfect gait (LS0) and especially in those that are in the acute phase of inflammation (LS1). It is precisely in these situations that the application of IRT is useful and can be considered a complementary diagnostic tool in the detection of lame cows because it can locate small but still significant temperature elevations and increase the accuracy in detecting diseased hooves, i.e., in detecting cows that have one or more diseased hooves.

Regarding the results presented in this article, it should be noted that a certain limitation is that it covers only one segment within comprehensive research that is still ongoing. A farm is presented, with its individual limitations; namely, the position and design of the milking parlor, which was an unsuitable location for positioning the IR camera, meaning the camera had to be positioned behind the milking parlor and in front of the corridor for the passage of cows, making automatic identification of cows impossible. We also tested the possibilities of IRT automation in different farm designs.

This research is the basis for further steps to come, such as more detailed statistical processing and the application of specific statistical models (ROC and GLM) that would calculate more precise values (sensitivity and specificity), and which would include other influences such as animal, order and stage of lactation, leg position, type of disease, etc.

In addition to the above, it is the basis for the development of new methods/applications for identifying cows in cases when the recording is made outside the milking parlor and when we cannot download their identification from the computer memory of the milking parlor.

High sensitivity to environmental factors (temperature, humidity, etc.), location of recording (indoors or outdoors) and recording performance itself (type of camera, camera position, camera settings, and cleanliness of hooves) makes it difficult to determine threshold values when applying IRT. For example, Werema et al. (2023) [39] determined a threshold value of 38.0 °C, while Werema et al. (2021) [42] determined an optimal threshold of 34.5 °C. In contrast to them, other authors determined much lower values: 23.3 °C [45], 25.5 °C, [43], and 27.0 °C [46]. In addition to large differences in threshold values, different authors determined large differences in reliability, such as the values of sensitivity of 78.5% and specificity of 39.2% determined by Lin et al. (2018) [45], while Rodríguez et al. (2016) [43] and Stokes et al. (2012) [46] found quite different values of sensitivity of 46.7% and 80% and specificity of 89.7% and 73%, respectively.

These are all challenges that must be considered when applying infrared thermography in the detection of hoof illnesses and lameness in dairy cows.

## 5. Conclusions

Considering the substantial daily demands on dairy farms, considerable efforts have been made to minimize manual labor through the implementation of automated lameness detection systems. The results obtained in this study provide another contribution to thermographic research by demonstrating that coronary band temperature distribution is influenced by hoof health status, ambient recording conditions, and lameness severity. The short period of 14 days in which the research was conducted represents a certain limitation, which is why it is necessary to continue with research. Therefore, the future research goal is to include multiple fixed effects (animal, leg position, and type of disease), and to refine temperature thresholds by collecting even more thermal values and lameness scores over a longer period of time at other locations (farms) with different environmental conditions. Although the practical implementation of automated systems has its challenges, the combination of different methods to increase the reliability and safety of hoof disease detection may improve the prevention of lameness in farm conditions in the future and, therefore, requires further development.

## Figures and Tables

**Table 1:** Descriptive statistic of the cows’ productive traits, infrared and microclimate measurements.

Traits	N	Mean	Min	Max	SD	CV	SE
PARITY	180 cows	2.98	1.00	8.00	1.18	39.73	0.04
DAYS IN LACTATION	180 cows	81.35	5.00	314.00	62.89	77.31	1.84
MILK PRODUCTION (kg/day)	180 cows	40.49	9.08	59.70	7.68	18.96	0.22
IR MEAN (°C)	1171 hooves	18.98	12.80	23.73	2.83	14.88	0.08
IR MAX (°C)	1171 hooves	20.70	13.30	27.49	2.92	14.12	0.09
IR MIN (°C)	1171 hooves	17.14	1.44	22.23	2.87	16.74	0.08
AIR TEMPERATURE (°C)	14 days	10.46	6.00	15.60	3.84	36.67	1.36
AIR HUMIDITY (%)	14 days	80.14	73.5	86.60	5.63	7.02	1.99

N—number of cases; SD—standard deviation; CV—coefficient of variation; SE—standard error of the mean; and IR—infrared.

**Table 2:** The 4-point manual locomotion scoring system according to DairyNZ (2023) [27].

Locomotion Score	Description
0 (Walks evenly)	The cow walks evenly with a confident walking speed similar to a person, long and regular strides, even weight bearing, a straight backline, and a steady head position.
1 (Walks unevenly)	The cow may have an uneven stride, a slightly arched back when walking, or a lowered head position.
2 (Lame)	The cow may walk slower, take shorter strides, bear weight unevenly, arch its back, and bob its head.
3 (Very lame)	The cow is very slow, stops often, barely stands on the lame leg, has an arched back, and large head movements.

**Table 3:** Temperature differences according to lameness score and different ambient conditions.

**HIGHER ambient condition**	**Lameness** **Score (LS)**	**N**	**IR MEAN**			**IR MAX**			**IR MIN**		
			**Mean**	**SD**	**SE**	**Mean**	**SD**	**SE**	**Mean**	**SD**	**SE**
	0	5	21.74 ^ns^	0.77	0.35	23.00 ^ns^	1.27	0.57	20.35 ^ns^	0.27	0.12
	1	22	21.89 ^ns^	0.80	0.17	23.42 ^ns^	1.11	0.24	20.30 ^ns^	0.64	0.14
	2	21	21.87 ^ns^	0.73	0.16	23.55 ^ns^	1.15	0.25	19.96 ^ns^	0.92	0.20
	3	19	21.88 ^ns^	1.48	0.34	23.40 ^ns^	1.70	0.39	20.02 ^ns^	1.35	0.31
	Total	67									
**LOWER ambient condition**	**Lameness** **Score (LS)**	**N**	**IR MEAN**			**IR MAX**			**IR MIN**		
			**Mean**	**SD**	**SE**	**Mean**	**SD**	**SE**	**Mean**	**SD**	**SE**
	0	6	16.28 ^a^*	1.116	0.46	17.98 ^a^*	1.63	0.66	13.87 ^a^**	0.58	0.24
	1	24	17.59 ^b^*	0.890	0.18	19.33 ^ab^	1.26	0.26	15.36 ^b^**	0.87	0.18
	2	24	17.71 ^b^*	1.097	0.22	19.65 ^ab^	1.45	0.30	15.58 ^b^**	1.15	0.24
	3	16	18.04 ^b^**	1.089	0.27	20.02 ^b^*	1.42	0.36	15.31 ^b^*	1.07	0.27
	Total	70									

N—number of cases; IR—infrared; SD—standard deviation; SE—standard error of mean; HIGHER—14.27 °C and 85.57% mean air temperature and humidity, respectively; LOWER—7.40 °C and 75.46% mean air temperature and humidity, respectively; ns = not significant; * *p* < 0.05; and ** *p* < 0.01 (different letters between lines indicate a significant difference).

## Data Availability

The data presented in this study are available on request from the corresponding author, as the data are part of an ongoing study.

## Abbreviations

The following abbreviations are used in this manuscript:

IRT	Infrared thermography
IR	Infrared
ICAR	International Committee for Animal Recording
IR MAX	The maximum infrared values
IR MEAN	The average infrared values
IR MIN	The minimum infrared values
CB	Coronary band
LS	Lameness score
LS0	Lameness score 0 (walks evenly)
LS1	Lameness score 1 (walks unevenly)
LS2	Lameness score 2 (lame)
LS3	Lameness score 3 (very lame)
